# The bone microstructure from anterior cruciate ligament footprints is similar after ligament reconstruction and does not affect long-term outcomes

**DOI:** 10.1007/s00167-021-06493-z

**Published:** 2021-02-20

**Authors:** Mateusz Stolarz, Jolanta Rajca, Paulina Cyganik, Jacek Karpe, Zygmunt Wrobel, Marcin Binkowski, Filip Humpa, Małgorzata Janik, Damian Czyzewski, Zbigniew Kwiatkowski, Krzysztof Ficek

**Affiliations:** 1 Department of Orthopedics and Traumatology, City Hospital in Zabrze, Zabrze, Poland; 2 Department of Computer Biomedical Systems, Institute of Computer Science, University of Silesia, Sosnowiec, Poland; 3 Galen-Orthopaedics, Bierun, Poland; 4 Department of Thoracic Surgery, Medical University of Silesia, Katowice, Poland; 5 Faculty of Physiotherapy, The Jerzy Kukuczka Academy of Physical Education in Katowice, Katowice, Poland

**Keywords:** Anterior cruciate ligament, ACL reconstruction, Bone tunnels, Tendon to bone healing

## Abstract

**Purpose:**

The purpose of this study was to assess the quality of the bone tissue microstructure from the footprints of the anterior cruciate ligament (ACL) and its impact on late follow-up outcomes in patients who undergo anterior cruciate ligament reconstruction (ACLR).

**Methods:**

The records of 26 patients diagnosed with a completely torn ACL who underwent ACLR were collected. During the surgery performed using the Felmet method, bone blocks from the native ACL footprints were collected. The primary measurements of the bone microstructure were made using a microtomographic scanner. In late follow-up examinations, a GNRB arthrometer was used.

**Results:**

There was no significant difference in the bone microstructure assessed using micro-CT histomorphometric data according to the blood test results, plain radiographs, age or anthropometric data. There was no difference in the bone volume/total volume ratio or trabecular thickness in the area of the native ACL footprints. Routine preoperative examinations were not relevant to the quality of the bone microstructure. The elapsed time from an ACL injury to surgery had no relevance to the results of arthrometry.

**Conclusion:**

The similarities in the microstructure of bone blocks from ACL footprints from the femur and tibia allow the variable use of these blocks to stabilize grafts in the Felmet method. The bone microstructure is not dependent on the time from injury to surgery. Histomorphometric values of the structure of the femoral and tibial ACL footprints have no impact on the long-term stability of the operated knee joint.

**Trial registration:**

The approval of the Bioethics Committee of the Silesian Medical Chamber in Katowice, Poland (resolution 16/2014) was given for this research.

**Level of evidence:**

II.

## Introduction

The healing process after anterior cruciate ligament reconstruction (ACLR) and the quality of the new connection between the bone and the ACL graft are not fully understood [8, 9]. Moreover, these characteristics are usually described based on animal models without considering many external factors, e.g., rehabilitation and chronic diseases. It is worth noting that the majority of scientific research focuses on the type of graft, attachment of the graft and means of enhancing healing [24].

Currently, the environment in which the graft is implanted, especially the bone layer of the bone tunnel, which directly adheres to the graft, is poorly characterized. Chronic diseases, the body mass index (BMI), and anthropometric data have a significant impact on the histomorphometric parameters of bone tissue [6, 19, 29]. Moreover, the internal environment of the bone tunnel is decidedly heterogeneous; it can be characterized by dividing it into three parts. The outer part includes the cortical bone of the diaphysis, the middle part consists of the trabecular bone, and the inner part consists of the cortex layer, which has direct contact with the joint cavity [1]. This heterogeneity has a biological effect on the speed of osseointegration of the graft [1] and the type of connection formed in different parts of the tunnel [26]. Lui et al. [14] noted that different parts of the bone tunnel exhibited significantly different environments, both biologically and mechanically, but the authors did not describe the structures.

The aim of this study was to assess the bone microstructure at the site of the femoral and tibial ACL footprints in patients who underwent ACLR. Our hypothesis assumes that the bone microstructure differs between the sites of femoral and tibial ACL attachment. The study also has the potential for indirect application in surgical practice by answering the following question: how soon after an injury should the surgery be performed?

## Material and methods

The Bioethics Committee of the Silesian Medical Chamber in Katowice, Poland (resolution 16/2014), approved this research. The study included 26 patients treated surgically due to total rupture of the ACL. All patients underwent surgery performed by the same orthopedic surgeon. The study group consisted of 7 women and 19 men. The patient age ranged from 18 to 59 years, with an average of 33 years (SD = 11.2) and a median of 30 years. All patients underwent the same preoperative diagnostic procedure, consisting of a physical examination, laboratory tests, and radiographic examination of the knee joint.

### Physical examination and laboratory tests

During the physical examination, the following tests were carried out: Lachman’s test, the anterior drawer test and the pivot shift test [3, 17]. For each of the patients, routine blood tests, electrolyte and glucose level determination, C-reactive protein (CRP) level determination and urinalysis were also performed.

### Radiographic imaging

In 21 patients, radiographs were routinely obtained preoperatively during admission to the hospital. The images were evaluated for possible fractures and intraarticular calcification. In the scans, the angle between the anatomical axes of the tibia and sagittal tarsus was determined in accordance with a previously described procedure [25]. The images were also evaluated for sclerotization of the subchondral layer of the tibial medial and lateral condyles. The level of sclerosis was not assessed.

### ACLR

All patients underwent ACLR. During the operation, the Felmet method (press-fit technique) was used [7]. Initially, hamstring muscle tendons—the semitendinosus and gracilis tendons—were collected as graft materials. The collected tendons were implanted in place of the previously damaged ligament. To attach the graft, bone tunnels were drilled in both the femur (femoral fixation) and the tibia (tibial fixation). The bone tunnel was prepared using cannulated drills, allowing bone blocks to be obtained. These blocks were used to attach the graft.

**Fig. 1:**
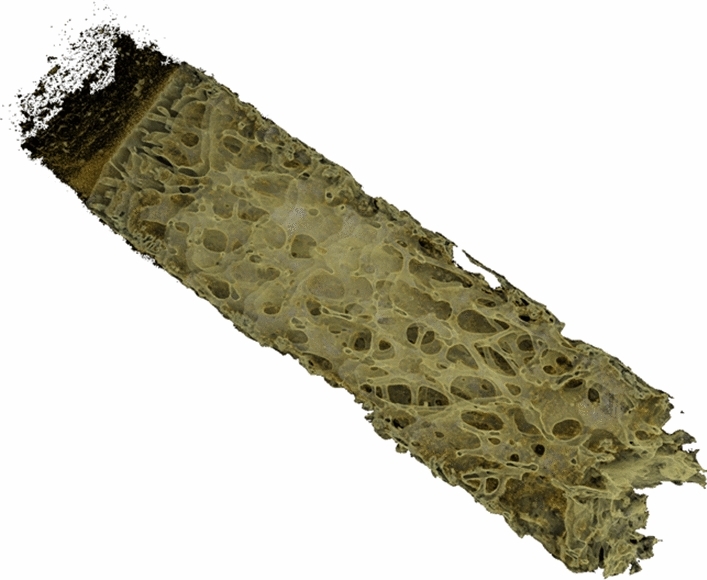
3D visualization of a bone block collected for testing during anterior cruciate ligament reconstruction (ACLR). Image obtained from micro-CT data reconstruction. The bone block contains bone tissue from the native ACL footprints

After surgery, some parts of the bone blocks remained. These were fragments of bone tissue from the attachment site of the native ACL (Fig. 1). Two bone blocks (samples) were collected from each patient: 1 from the femur and 1 from the tibia. These residues were subjected to qualitative and quantitative analysis to obtain objective data on the quality of the bone tissue.

### Micro-CT imaging

The GE Measurement and Control microtomographic (micro-CT) scanner was used for the study. The following parameters were used to scan the femoral and tibial bone blocks: 120 kV, 100 mA, and Cu 0.1. One thousand projections were made, representing 2D images of each tested sample stored in 16-bit Tagged Image File Format (tiff) with individual image dimensions of 2048 × 2048 pixels, corresponding to the dimensions of the detector used. On the basis of the projection image collection, in the tomographic reconstruction process, cross-sectional images of the sample were obtained. A single voxel of the resulting cross-sectional image was 20.072 × 20.072 × 20.072 µm. The microtomographic data obtained from the femoral and tibial bone blocks were then subjected to further analysis. Two sets of images were generated from each sample: one for the trabecular bone and one for the cortical bone. Bone measurements were performed using ImageJ software [23] with the BoneJ plugin [5]. Drishti (an open-source 3D volume rendering software) [13] was used to visualize micro-CT data. Figure 1 shows the 3D model of a bone block collected for testing during ACLR.

Samples were scanned with a hydroxyapatite (HA) phantom (containing five cylinders of various HA crystal concentrations). Gray density (GD) values were calculated in correlation with the HA density (g HA/cm^3^), and calibration curves for each sample were obtained. The use of a calibration curve allows quantitative determination of the bone mineral density (BMD).

**Fig. 2:**
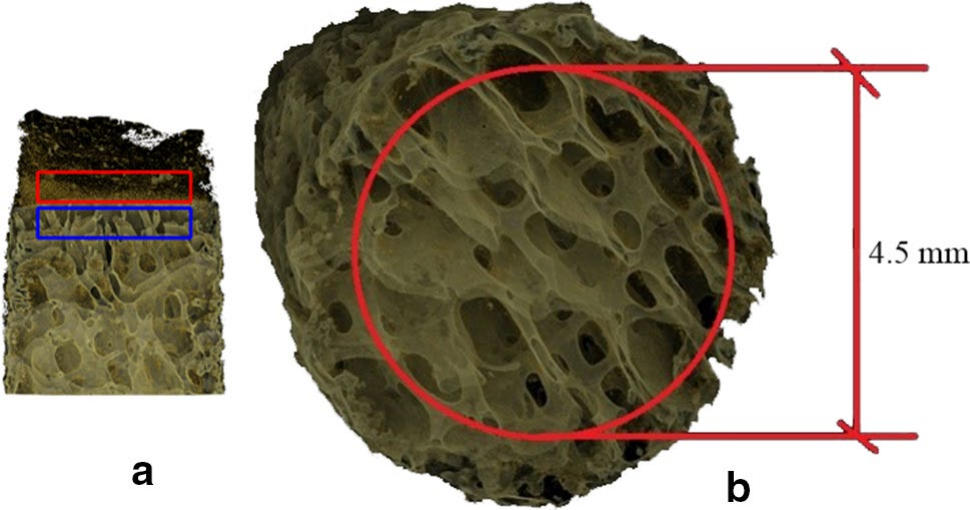
The region of interest (ROI) of the image is marked. The ROI contains bone tissue from the native ACL footprints. **a** Red ROIs include cortical bone, and blue ROIs include trabecular bone. The area of 30 subsequent cross-sections was examined. **b** The cross-section ROI included the area of a circle with a diameter equal to 4.5 mm in the middle part of the bone (without edges that may generate errors due to the unfavorable boundary effect)

On the axial cross-sections, the regions of interest (ROIs) for the femoral and tibial bone blocks were selected, including cortical and trabecular bone separately (Fig. 2a). The ROI included an area inside a circle with a diameter of 4.5 mm (Fig. 2b) through 30 subsequent slices for cortical and trabecular bone. Based on the selected ROIs, the BMD and histomorphometric parameters were calculated separately for tibial cortical bone (TC), tibial trabecular bone (TT), femoral cortical bone (FC), and femoral trabecular bone (FT), including the following: the bone volume fraction (BV/TV)—the volume of mineralized bone per unit volume of the sample, [%]; the trabecular thickness (Tb.Th)—the mean arithmetic thickness of trabecular bone, [mm]; the degree of anisotropy (DA)—the level of structural anisotropy; the connectivity—the number of connections between the individual elements of a structure; and the structure model index (SMI)—an indicator describing the plate- or rod-like geometry of trabecular structures (SMI = 0 for plates, 3 for rods).

### Follow-up and arthrometry examinations

**Fig. 3:**
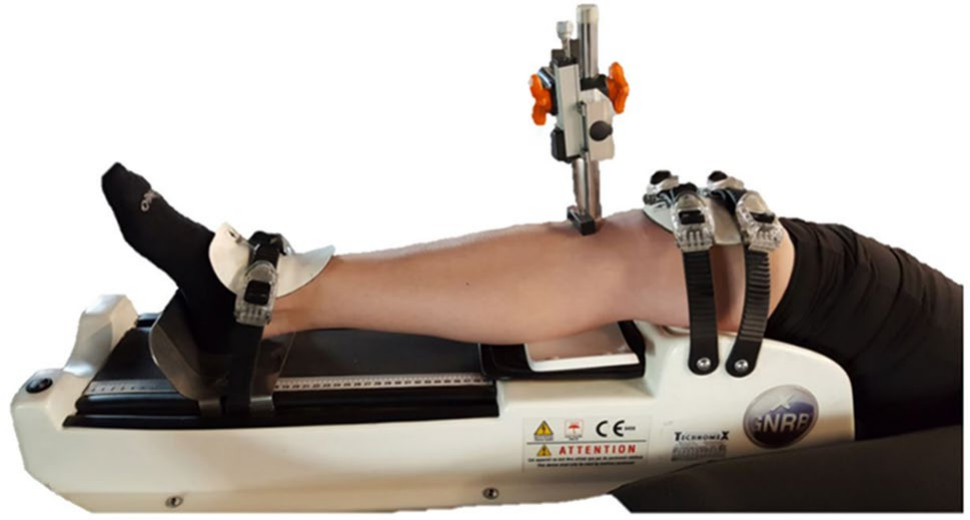
An example of a GNRB arthrometry examination with lower limb placement for arthrometry. The examination was conducted as a follow-up. The healthy and operated knees were compared

At 1–1.5 years after ACLR surgery, patients were subjected to routine control follow-up examinations. Sixteen patients were scheduled for a control visit (16 patients were lost to follow-up). Orthopedic tests (Lachman’s test, the anterior drawer test, and the pivot shift test) were repeated during the follow-up period. Additionally, the function of the reconstructed ACL was assessed using a GNRB® arthrometer (Genourob, Laval, France) (Fig. 3). Measurements of knee laxity were performed using the following settings: 134 N, 160 N, and 200 N [20].

### Statistical analysis

The statistical analysis was performed using Statistica software (release 12.0, StatSoft). The normality of data distributions was checked by the Kolmogorov–Smirnov test. Student's t test was used to compare normally distributed data between study groups, and the results are presented as the mean, minimum, maximum, and standard deviation. For nonnormally distributed data, the Mann–Whitney *U* test was used, and the results are presented as the median, minimum, maximum, and interquartile range. Differences with *p* < 0.05 were considered statistically significant. The sample size was determined based on a power analysis (G*Power 3.1.9.4); in the analysis, the following parameters were applied: test family = *t* test, power (1 − *β*) = 0.8, effect size *d* = 0.7, and *α* = 0.05.

## Results

ACLR contributed to a reduction in knee joint instability in each patient. Five individuals reported periodic pain in the operated area, particularly during weather changes. Among the studied patients, two patients experienced prolonged knee swelling, which had no effect on the stability or the treatment outcome. None of the patients suffered damage to the transplanted ligament.

### Histomorphometric analysis

**Fig. 4:**
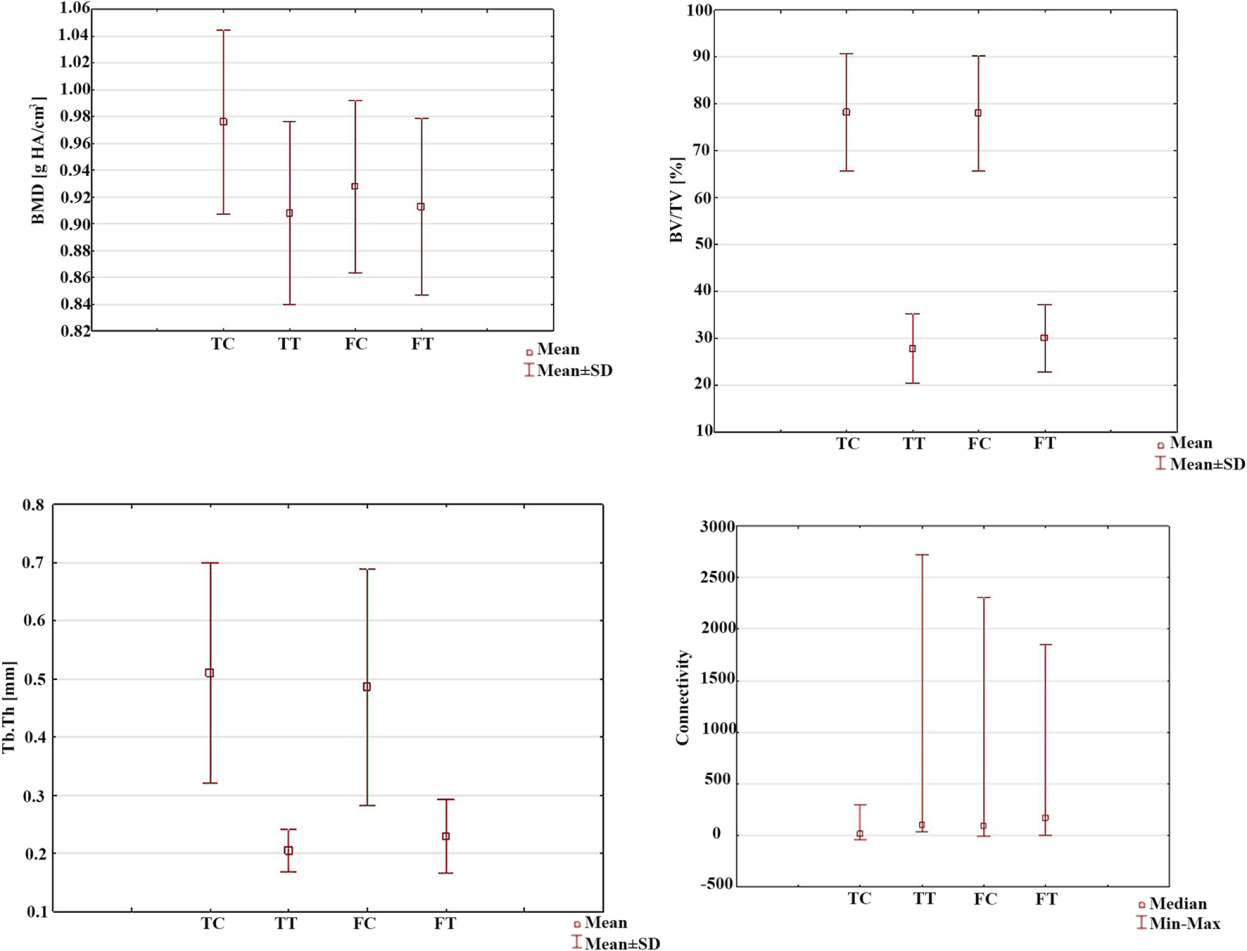
Comparison of the micro-CT data, including the bone mineral density (BMD), bone volume fraction (BV/TV), trabecular thickness (Tb.Th), and connectivity, between groups. *TC* tibial cortical bone, *TT* tibial trabecular bone, *FC* femoral cortical bone, *FT* femoral trabecular bone

Comparisons of the bone structure parameters between TC, TT, FC and FT are provided in Fig. 4 and Tables 1, 2, 3, 4, 5. The following significant differences were found between groups: TC-TT (*p* = 0.001) and TC-FC (*p* = 0.015) for BMD; TC-TT (*p* < 0.0001) and FC-FT (*p* < 0.0001) for BV/TV; TC-TT (*p* < 0.0001) and FC-FT (*p* < 0.0001) for Tb.Th; TC-TT (*p* < 0.0001), TC-FC (*p* = 0.001) and FC-FT (*p* = 0.043) for connectivity; and TC-TT (*p* < 0.0001) and FC-FT (*p* < 0.0001) for SMI.

**Table 1:**
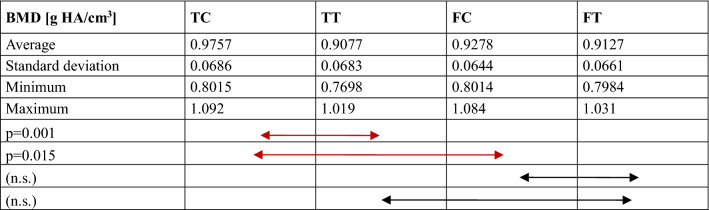
Comparison of the bone mineral density (BMD)

*TC* tibial cortical bone, *TT* tibial trabecular bone, *FC* femoral cortical bone, *FT* femoral trabecular bone, *n.s.* no significance

**Table 2:**
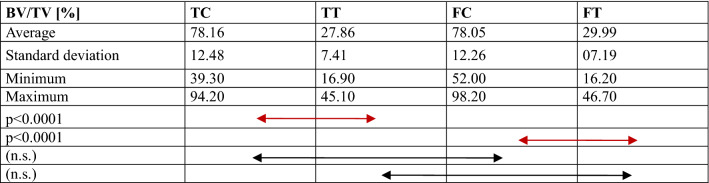
Comparison of the bone volume fraction (BV/TV)

*TC* tibial cortical bone, *TT* tibial trabecular bone, *FC* femoral cortical bone, *FT* femoral trabecular bone, *n.s.* no significance

**Table 3:**
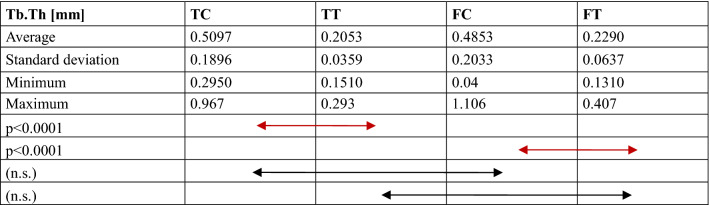
Comparison of the trabecular thickness (Tb.Th)

*TC* tibial cortical bone, *TT* tibial trabecular bone, *FC* femoral cortical bone, *FT* femoral trabecular bone, *n.s.* no significance

**Table 4:**
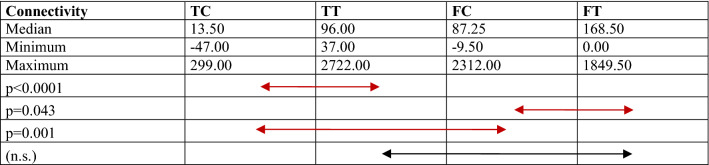
Comparison of the connectivity

*TC* tibial cortical bone, *TT* tibial trabecular bone, *FC* femoral cortical bone, *FT* femoral trabecular bone, *n.s.* no significance

**Table 5:**
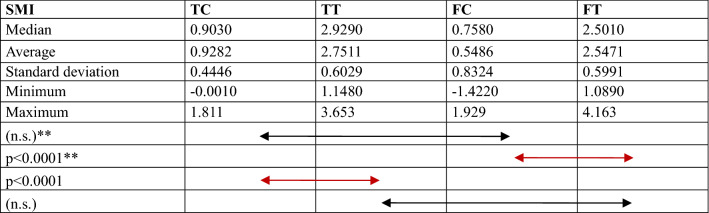
Comparison of the structure model index (SMI)

*TC* tibial cortical bone, *TT* tibial trabecular bone, *FC* femoral cortical bone, *FT* femoral trabecular bone. **—Mann–Whitney *U* test, *n.s.* no significance

### Correlations between data

**Fig. 5:**
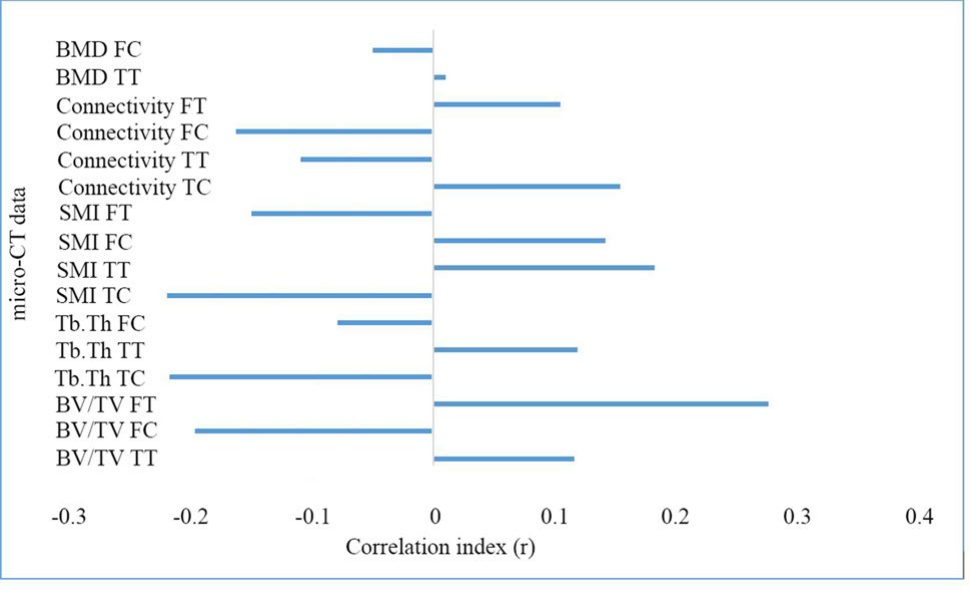
Correlation coefficients between the time from injury to ACLR [weeks] and histomorphometric data obtained by micro-CT

The Pearson correlation coefficient was calculated to measure the linear dependence between data. The results showed no significant correlation between histomorphometric parameters and the following: (1) blood and urine test results; (2) findings of plain radiographs of knee joints; (3) patient weight, height or age; and (4) the time from injury to surgery (Fig. 5).

**Fig. 6:**
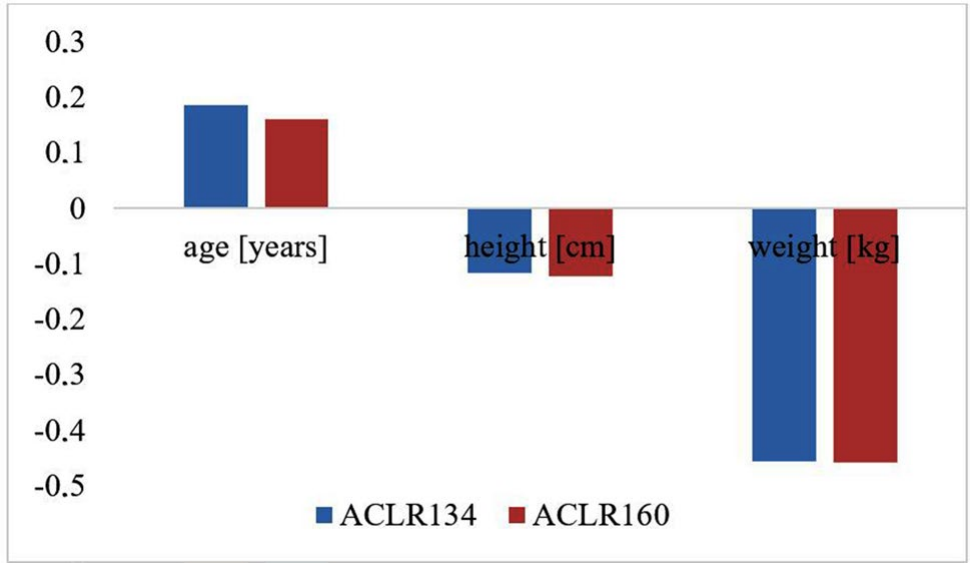
Correlation coefficients of age [years], weight [kilograms] and height [centimeters] with arthrometric data over 1–1.5 years of follow-up. ACLR134, applying a force = 134 [N]; ACLR160, applying a force = 160 [N]

The correlation coefficients between the time from injury to surgery, calculated in weeks, and arthrometric data (ACLR134 and ACLR160) were also determined. The significance of correlation coefficients between the abovementioned parameters was also not confirmed. Figure 6 shows the values of the correlation coefficients of age, weight and height with the arthrometric data. The significance of the correlation coefficients between the abovementioned parameters was not confirmed.

The results of arthrometry were significantly inversely proportional to the “connectivity” for the cortical and trabecular bone of the tibia and for the trabecular bone of the femur of the nonoperated knee joint (Table 6). This relationship was not confirmed for the knee joint after ACLR. The values of connectivity varied in the knee joint after ACLR regardless of the force used in arthrometry. Other histomorphometric data, including the BMD, BV/TV, Tb.Th, and SMI, did not show a significant relationship with the arthrometry results.

**Table 6:** Comparison of the correlation coefficients of connectivity with arthrometric data

Connectivity	NO134	NO160	NO200	ACLR134	ACLR160	ACLR200
TC	− 0.716	− 0.6996	− 0.7201	− 0.0464	− 0.0063	0.0354
	**p = 0.020**	**p = 0.024**	**p = 0.019**	(n.s.)	(n.s.)	(n.s.)
TT	− 0.677	− 0.6522	− 0.6615	0.054	0.1004	0.1479
	**p = 0.032**	**p = 0.041**	**p = 0.037**	(n.s.)	(n.s.)	(n.s.)
FC	− 0.6665	− 0.6572	− 0.6511	− 0.1701	− 0.1285	− 0.0906
	**p = 0.035**	**p = 0.039**	**p = 0.041**	(n.s.)	(n.s.)	(n.s.)
FT	− 0.2627	− 0.2487	− 0.2767	0.3625	0.4115	0.4668
	(n.s.)	(n.s.)	(n.s.)	(n.s.)	(n.s.)	(n.s.)

*TC* tibial cortical bone, *TT* tibial trabecular bone, *FC* femoral cortical bone, *FT* femoral trabecular bone. NO—nonoperated knee joint; ACLR—knee joint after ACLR; 134, 160, and 200 are values of subsequent forces used during the test, expressed in [N]; *n.s.* no significance

## Discussion

The most important finding of the present study was the lack of a difference in bone microstructure between the tibial and femoral bone blocks, which suggests the possibility of using bone blocks interchangeably during ACL graft fixation in the Felmet method. To the best of our knowledge, this is the first such in-depth assessment of the bone microstructure among patients undergoing the ACLR procedure. At the time of writing, no similar research in human subjects was available in the literature; the presented research is pioneering and prospective. To date, no human bone tissue has been examined in the context of ACL grafts using micro-CT. The group of assessed patients consisted of 26 individuals, which is a relatively large study group compared to that in previous studies evaluating the bone tunnel using micro-CT. In studies on similar subjects, most study groups consisted of fewer than 26 animals [1, 14, 16], and some were as large as 28 animals [28]. It should be noted that the study group of patients was heterogeneous in terms of age, sex, and side of the operated joint. The basis of qualification for inclusion in the study was a total rupture of the ACL. Lack of homogeneity did not affect the results of the assessments of the function of the knee by follow-up arthrometry. Arthrometry assessment of the knee allows for assessment of the degree of ACL failure after trauma and total instability. The examination facilitates the patient's qualification for surgical or conservative treatment. Performing measurements after ACLR enables monitoring of the capacity of the ligament graft and stability of the knee in subsequent stages of rehabilitation.

Before surgery, the majority of patients underwent radiographic examination of the knee joint in the anteroposterior (AP) and lateral projections. The literature describes significant relationships between features on radiographic examinations and histomorphometric data obtained by micro-CT [11, 15]. In a tibial cadaveric study, Hirvasniemi et al. [11] compared radiographs with micro-CT images and found a significant relation between the two examination methods for bone density and structure. In the presented studies, there were no statistically significant relationships between the radiographic data and histomorphometric parameters calculated based on micro-CT images. However, it should be noted that the examined patients did not have chronic diseases. In addition, radiographic images were analyzed for sclerosis by a radiologist, and computerized analysis was not used for this purpose.

Analysis including the anthropometric data of patients, such as weight, height and age, showed no significant impact on the bone microstructure, despite the expectation of a relationship between BMI and bone quality, including mineral density. Evans et al. [6], in densitometry and tomography studies in a group of 100 people, showed that obese adults had a higher bone density and thicker trabeculae than adults with a normal body weight. The authors claimed that obesity can protect against age-related bone loss and may increase the peak bone mass. In the conducted analyses, there were no statistically significant results indicating a relation of age, weight or height with bone microstructure. However, the studied group of patients was significantly smaller than that in the cited studies.

The results of blood tests did not show a significant correlation with the bone microstructure or arthrometry outcomes. These results suggest a lack of influence of blood test parameters in healthy people on the quality of bone tissue and long-term results of treatment after ACLR.

Micro-CT has been repeatedly used to assess the bone tunnel during healing of the ligament [14, 27, 28]. The histomorphometric analysis showed that the BV/TV ratio was significantly higher in the cortical bone than in the trabecular bone of both the tibia and femur. This result confirms the structure of the bone tissue [14, 22]. This finding also indicates that the bone tissue at the site of native ACL attachment is typical, as described in the literature [22]. There was no difference in BV/TV of the blocks, suggesting the possibility of replacing the femoral and tibial blocks to fix the ligament.

Another parameter considered was the arithmetic mean of the thickness of trabeculae (Tb.Th). The trabecular thickness is directly related to the ratio of the volume of bone tissue to the test sample volume (BV/TV). Thicker bone bars, indicated by larger BV/TV values, are present in the cortical bone layer. Thinner bone bars characterize trabecular bone. The lack of differences in the thickness of the trabecular bone in the bone tissue surrounding the native ACL attachments to the femur and tibia, as indicated by the BV/TV ratio, indicates the possibility of using replacement bone blocks in ACLR surgery.

The number of connections between individual elements of a given structure is determined by the connectivity parameter. It is an independent parameter describing bone tissue with a “weak dependence” on the BV/TV [18]. Research has shown that the connectivity is directly proportional to the stiffness and strength of bone tissue [12, 18]. These values ​​decrease, for example, in osteoporosis. The results of our research are similar to previously reported results [18], showing a statistical significance between particular layers of the tibia and femur. In the presented studies, the arthrometry results were significantly inversely proportional to the connectivity for the cortical and trabecular layers of the tibia and to the cancellous layer of the femur of the nonoperated knee joint (Table 6). This correlation was not confirmed for the knee after reconstruction of the ACL.

A significant correlation was found between the SMI of the trabecular and cortical bone, indicating the previously mentioned possibility of using bone blocks interchangeably during ACL graft fixation. However, it should be noted that in light of recent studies, the SMI should not be used for the quantitative assessment of bone tissue. Salmon et al. [21] found that SMI is negative in the case of concave surfaces that are common in the trabecular bone in various studies. This was confirmed in the present study, where some of the minimum values obtained were negative. In conclusion, the SMI is not a useful tool for quantifying the actual bone structures used during ACLR.

Using micro-CT, Wen et al. [27] studied changes that occur in the bone tissue of rabbits at the bone-graft interface at various intervals after ACLR. While assessing changes in the bone microstructure, the authors noticed that the environment in which the graft was deposited was initially characterized by a loss of bone mass, especially in the tibia. Over time, the values of morphometric parameters increased. This should raise concerns about the safety of bone graft transplantation in clinical practice and require targeted research to improve the environment and the quality of healing. This support for bone fixation was assessed using micro-CT by a similar team of scientists [28]. Studies conducted in rabbits confirmed that calcium-phosphate cement instead of a bone-graft interface supports the osseointegration of soft tissues and improves the quality of the bone structure around the bone tunnel during healing. This fact confirms the structural diversity of the bone, depending on the location of the test layer and individual variations.

The BMD is related to the content of essential mineral salts, which are responsible for the hardness, stiffness and resistance to deformation of bone [22]. The BMD is particularly important in the identification of osteoporosis and osteopenia. The literature search did not yield any studies on the influence of the BMD on the outcome of the surgical treatment of a damaged ACL and the bone microstructure. The presented outcomes cannot provide answers as to whether differences in bone density affect the long-term results after ACLR. Statistically significant results for the BMD of the tibial and femoral cortex layers are related to the transferred loads. Higher BMD values ​​were noted in the tibia, at the site of ACL attachment, which lies closer to the areas of heavier loads. Lower values were found in the femur at the site of native ligament attachment, which lies further from the surface and is loaded the same as the epiphysis.

An issue worthy of special attention is the time from injury to ACLR. There is no direct guidance in the literature regarding the optimal time to perform ACL reconstruction after injury. Chhadia et al. [4] concluded that an extended time prior to surgery is closely related to an increased risk of medial meniscus injury and joint cartilage damage. Granan et al. [10] reported that the chances of damage to the medial meniscus following damage to the ACL increased by 1% for every month spent waiting for ligament reconstruction, and damage to the articular cartilage occurred twice as often if the meniscus had been damaged. On the other hand, after damage to the ACL, proinflammatory cytokines are released into the knee joint cavity. Bigoni et al. [2] investigated the biochemical composition of synovial fluid in patients after ACL injury; this study showed that within a short time after injury, proinflammatory cytokines accumulated, particularly IL-6 and IL-8. These cytokines can exacerbate degenerative changes in the articular cartilage.

In the present study, the time from injury to ACLR had no direct effect on histomorphometric parameters determining the quality of the bone microstructure from ACL footprints. Moreover, the time from injury to ACLR did not affect the results of the arthrometric examination of the operated knee, performed for more than a year after surgery. In summary, in the context of the literature review and the results of the presented studies, immediate reconstruction after ACL injury is recommended.

With regard to clinical relevance, the findings of the current study will help surgical specialists at the time of damage to choose one of the bone blocks during the collection procedure because the second block can be used to provide similar healing. Furthermore, the authors recommend immediate reconstruction surgery after the ACL injury.

A limitation of this study was the number of patients who completed the follow-up visit (only 16 of 26 participants) 1 year after ACL surgery; therefore, arthrometry was performed only in those patients. Second, image segmentation was performed by only one experienced biomedical engineer; therefore, the interrater variability could not be determined. Additionally, because the bone blocks differed in size, bone structure measurements were obtained in a limited volume in both trabecular and cortical bone. Future work should focus on obtaining bone structure measurements in a larger group of patients as well as in a larger volume in bone blocks.

## Conclusion

In conclusion, it was confirmed that micro-CT is an accurate and safe method for assessing the bone microstructure. A reliable assessment of the results of preoperative examinations, including routine blood tests, radiography, and anthropometry, as well as age, revealed no significant effect on the bone microstructure. Studies show that the bone microstructure is not dependent on the time from the ACL injury to ACLR surgery. In this context, taking into account the additional damage to the meniscus and cartilage confirmed by reports in the literature, it is worth performing the procedure as soon as possible. Histomorphometric values of the structure of the femoral and tibial bone have no impact on the long-term stability of the operated knee joint.
